# Evaluation of Spirometric Measures and Quality of Sleep in Tuberculosis Patients and Their Non-Tuberculosis Family Caregivers

**DOI:** 10.7759/cureus.17788

**Published:** 2021-09-07

**Authors:** Afreen Begum H Itagi, Satish P Dipankar, D Krishna Veni, G Y Yunus

**Affiliations:** 1 Physiology, All India Institute of Medical Sciences, Mangalagiri, IND; 2 Physiology, All India Institute of Medical Sciences, Patna, IND; 3 Physiology, Nimra Institute of Medical Sciences, Vijayawada, IND; 4 Public Health Dentistry, ESIC Dental College, Kalaburagi, IND

**Keywords:** tuberculosis, quality of sleep, morbidities, family contacts, pulmonary functions.

## Abstract

Background and Aim

Tuberculosis (TB) patients may present with depression and poor sleep as co-morbidities. This presents great challenges including the stigma of increased risk of developing TB while taking care of such patients. This study aims to determine the lung functions, quality of sleep in tuberculosis patients in comparison with non-tuberculosis (non-TB) family caregivers.

Methods

TB patients and their family caregivers (60 each) visiting the Directly Observed Therapy Short-course (DOTS) clinic at a tertiary care hospital were assessed for spirometric parameters and quality of sleep. Spirometry measurements were performed using a portable, computerized, pre-calibrated, electronic, dry type of machine. Pulmonary function impairment pattern and severity were assessed from spirometry results using a percentage of the predicted values of Forced Vital Capacity (FVC) and Forced Expiratory Volume in one second (FEV_1_). Descriptive statistics and t-tests were applied using SPSS version 19.0. p≤0.05 was considered significant.

Results

TB patients had a significantly more (p=0.000) sleep disturbance, daytime dysfunction, and higher mean global Pittsburgh Sleep Quality Index (PSQI) score (9.56±3.97) compared to their non-TB family caregivers (4.36±2.07). The spirometric measures were reduced in TB patients and showed significant differences in actual measures of all parameters except FVC (actual). The % predicted measures of FEV_1_, FEF _25%-75%_, PEFR, and MVV showed significant differences in comparison to their non-TB family caregivers.

Conclusions

The present study shows that TB patients have a poor quality of sleep and pulmonary functions compared to their non-TB family caregivers. Health care workers need to develop systematic strategies to screen the symptoms of mental disorders in tuberculosis patients and their family caregivers to enable better management of this population.

## Introduction

India accounts for 27% of the world’s Tuberculosis (TB) cases and is ranked first in the World Health Organisation (WHO) list of countries with the highest TB burden [1, 2]. Many studies have shown that pulmonary tuberculosis can lead to obstruction of airflow. Longitudinal studies have shown that a significant percentage of patients with treated pulmonary TB show evidence of permanent airflow obstruction or restrictive impairment [3,4]. Post-tuberculosis pulmonary dysfunction has therefore emerged as a distinct clinical entity, which subsequently impairs the quality of life of patients [5]

Tuberculosis patients suffer from co-morbid depression and anxiety, which ultimately may lead to sleep deprivation, hence affecting the physical and/or cognitive function and thus jeopardizes the quality of life [6,7]. Several studies have postulated the prevalence of co-morbid depression and anxiety among tuberculosis patients, but there exists a dearth of literature associated with the sleep quality among TB patients [8,9,10]. Family contacts exposed to TB patients are at greater risk of contracting TB than the general population [11]. The perception of TB stigma and fear of acquiring infection are also associated with serious socioeconomic consequences [12].

There are not many mental health studies on TB contacts conducted in the past and whether family caregivers are at risk of psychological distress further remains unclear. However, it can be hypothesized that family caregivers of TB patients may develop depressive disorders and hence sleep deprivation due to concern for the risk of TB infection in themselves and possible potential socioeconomic stress if the index cases of TB are their family members or people who live with them. This observational study aims to assess the quality of sleep and pulmonary function parameters among tuberculosis patients and their family caregivers visiting a tertiary care health facility.

## Materials and methods

This cross-sectional study was undertaken from Jan 2020 to June 2020 at the Directly Observed Therapy Short-course (DOTS) clinic of the Medical Outpatient Department / DOTS clinic of Siddhartha government medical college and tertiary health care facility in Andhra Pradesh, India. Patients with pulmonary TB above 18 years of age, attending DOTS clinic and who consented to participate in the study served as cases. The comparison group was taken as accompanying family members who consented to take part in the research.

The sample size determination was carried out by using Gpower version 3.1.9.7 for Windows, with an effect size of the study at 0.7 (medium; desired = <1), keeping the power of the study as 0.95 (95%) and significance level (p-value) at 0.05. The estimated minimum sample was 55 in each group. A total sample size of 120 subjects (60 in each group) were included in the study after obtaining written informed consent in accordance with the Institutional Review Board approved protocol after being explained the purpose and procedures in detail. Ethical approval was obtained before conducting the research, from the Institutional ethical committee (IEC/AIIMS/Mangalagiri/2020-21/10). A self-administered questionnaire was designed and pretested in both Telugu and English languages for ensuring comprehension by all subjects. Adult patients with pulmonary tuberculosis (TB) attending DOTS clinic and their apparently healthy adult family members who consented to participate in the study were included. TB patients or family members with pre-existing asthma/ chronic obstructive pulmonary disease (COPD), paraplegia, heart failure, stroke, extrapulmonary tuberculosis, spine deformities (kyphosis, scoliosis), chest deformities, obstructive sleep apnea, pregnancy, and subjects undergoing treatment for depression prior to start of the study were excluded. 

Quality of sleep

A self-rated, 21 item Pittsburgh Sleep Quality Index (PSQI) questionnaire consisting of seven components: subjective sleep quality, sleep latency, sleep duration, sleep efficiency, sleep disturbances, use of sleep medications, and daytime dysfunction was administered to evaluate sleep quality in all the participants. Each item in the PSQI was scored from 0 to 3, and the PSQI global score was the sum of these items (range: 0 to 21), where higher scores indicate worse sleep quality. Poor sleep quality was defined as a PSQI score >5; as a global PSQI score >5 is shown to have a diagnostic sensitivity and specificity and is used to separate good sleepers (≤5) from poor sleepers (>5) [13].

Spirometric assessment

The spirometry assessment was performed using a portable, computerized, pre-calibrated, electronic, dry type of machine - “NDD - Easy on PC” (Royal Medi systems). Spirometry was done using standard TB precautions; disposable mouthpiece/filter (spirette/filter) per participant were used to avoid cross-contamination. The forced expiratory manoeuvres were explained to the participants before they underwent spirometry. The subjects were to sit comfortably and relaxed in an armed chair with straight back and were asked to inhale atmospheric air deeply. The nose clip was placed immediately and keeping the spirette inside the mouth with lips tightly sealed around it, the subject was asked to blow out air as fast and as hard as possible, blast out for a minimum of 6 seconds, followed by the inhalation deeply with the spirette still inside the mouth (to form a loop). Best of 3 trials performed with an interval of 5 minutes was taken for analysis. The forced vital capacity (FVC), forced expiratory volume in one second (FEV_1_), mean forced expiratory flow during the middle of FVC (FEF_25% -75%_), peak expiratory flow rate (PEFR), and maximum voluntary ventilation (MVV) were measured. Lung function impairment pattern and severity were assessed from spirometry results using percentage of the predicted values of FEV_1_/FVC and *FVC*

Statistical analyses were performed using SPSS version 19 (IBM SPSS, Chicago, USA). Kolmogorov-Smirnov test was used for determination of the data distribution. All characteristics were summarized descriptively. For continuous variables, summary statistics of N, mean, standard deviation about the arithmetic mean were used. For categorical data, frequency and percentage were used. The difference in parameters between the two groups was analyzed using an unpaired/independent t-test. At a confidence interval of 95%, the test was considered “highly significant” if it yielded p < 0.001. p-value ≤ 0.05 is taken as 'significant'.

## Results

**Figure 1:**
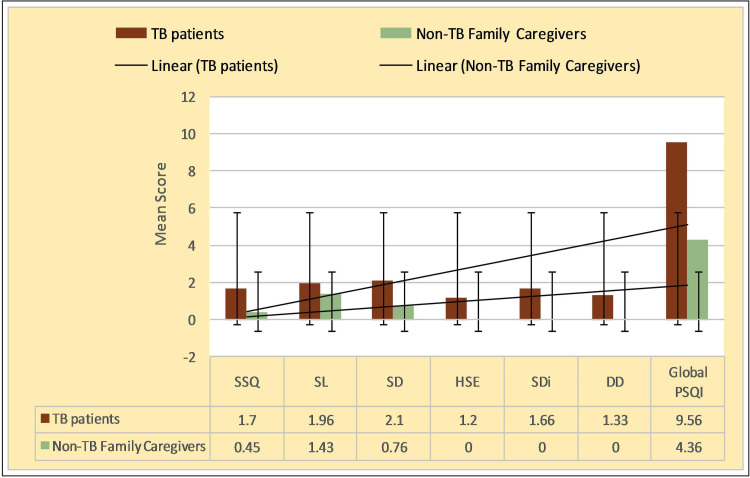
Quality of sleep among the TB patients and non-TB.family caregivers SSQ = Subjective Sleep Quality score, SL = Sleep Latency score, SD = Sleep Duration score, HSE =  Sleep Efficiency Score, SDi = Sleep Disturbance component score, DD = Day time Dysfunction score.

The distribution of the TB patients and their accompanying non-TB family caregivers (n=60 each) according to their socio-demographic characteristics are as shown in Table 1. TB patients had a significantly higher mean global PSQI score compared to their non-TB family caregivers (p= 0.000). Similarly, subjective sleep quality, sleep duration, sleep disturbance, and daytime dysfunction were also significantly more in TB patients, indicating a poorer sleep quality (p= 0.000). However, the PSQI sleep latency mean score among TB patients was high but with a comparatively lesser difference score among non- tuberculosis (non-TB) caregivers (p<0.05) (Figure 1).

**Table 1:** Socio-demographic characteristics of participants. NA- not applicable

Characteristics		TB patients		Non-TB family caregivers	
		No.	%	No.	%
Age	≤24	04	6.67	10	16.67
	25–44	25	41.67	22	36.67
	45–59	23	38.33	26	43.33
	≥60	08	13.33	02	3.33
Sex	Male	46	76.67	24	40.0
	Female	14	23.33	36	60.0
Marital Status	Married	54	90.0	50	83.33
	Single	06	10.0	08	13.33
	Separated /Widowed	00	00	02	3.33
Education	No formal education	00	00	00	00
	Primary education	08	13.33	12	20.0
	Secondary and above	52	86.67	48	80.0
Occupation	Employed	00	00	00	00
	Private	28	46.67	18	30.0
	Student	00	00	06	10.0
	Farmer/Daily laborers	26	43.33	20	33.33
	Unemployed	06	10.0	16	26.67
Family Size	1–2	00	00	00	00
	3–5	42	70.0	42	70.0
	More than 5	18	30.0	18	30.0
TB treatment duration	< 3months	10	16.67	NA	NA
	3–6months	32	53.33	NA	NA
	> 6months	18	30.0	NA	NA

Lung function measures were reduced in TB patients and the difference was significant compared to their non-TB family contacts (Table 2). Predicted mean FVC, FEV_1_, FEV_1_/FVC ratio, FEF_25-75%_, PEFR, and MVV were analyzed for both TB patients and their non-TB family contacts. Values for all measurements are expressed as mean (%) ± SD (Table 2). Mean FVC and FEV_1_/FVC of TB patients were lower compared to non-TB family contacts; however, this difference was not statistically significant. Similarly, it was found that mean FEV1, FEF_25-75%_, and PEFR of TB patients were significantly lower than that of their non-TB family contacts (Table 2). A significantly higher mean of MVV was observed among non-TB family contacts as compared to TB patients.

**Table 2:** Comparison of spirometric measures among the TB patients and their non-tuberculosis (non-TB) Family caregivers SD: standard deviation;  FVC - Forced vital capacity; FEV1- Forced expiratory volume in one second; FEF_25-75%_ - Mean forced expiratory flow during the middle of FVC; PEFR- Peak expiratory flow rate; MVV - Maximum voluntary ventilation ; % pred = percentage predicted value; *p Value ≤ 0.05 =  'Significant'

Parameter	TB patients	Non-TB Family caregivers	Mean Difference	t Value	Sig (p-Value)
	Mean± SD	Mean± SD			
FVC_Predicted	3.043± 0.73	2.970± 0.57	0.07±0.12	0.60	0.55
FVC_Actual	2.002± 0.99	2.213± 0.90	0.21± 0.17	-1.218	0.226
FVC (% pred)	65.20± 26.5	72.20± 19.1	7.0±4.22	-1.658	0.100
FEV1_Predicted	2.533± 0.61	2.543± 0.48	0.01±0.10	-0.100	0.098
FEV1_ Actual	1.485± 0.56	1.824± 0.67	0.33±0.11	-2.983	0.003*
FEV1 (% pred)	57.73±21.5	69.53± 15.8	11.8±3.45	-3.42	0.001*
FEV1/FVC_ Predicted	82.35±1.45	85.68± 3.05	3.33±0.43	-7.621	0.001*
FEV1/FVC_ Actual	76.38±18.9	83.13± 11.6	6.75±2.86	-2.357	0.02*
FEV1/FVC (% pred)	92.93±23.3	98.11± 12.8	5.18±3.44	-1.505	0.135
FEF_25-75%__ Predicted	3.116±0.69	3.286± 0.61	0.17±0.11	1.432	0.155
FEF_25-75%__ Actual	1.415±1.44	1.869± 0.81	0.45±0.21	2.122	0.036*
FEF_25-75% _(% pred)	38.76±21.9	56.05± 19.8	17.28±3.81	4.526	0.001*
PEFR_ Predicted	7.264±1.14	6.589± 1.16	0.67±0.21	3.198	0.002*
PEFR_ Actual	2.344±1.08	2.873± 1.24	0.53±0.21	-2.482	0.014*
PEFR (% pred)	31.23±12.7	43.03± 14.5	11.85±2.49	-4.757	0.001*
MVV_ Predicted	99.40±22.9	97.43± 16.5	1.96±3.65	0.539	0.591
MVV_ Actual	50.48±24.4	58.13± 22.1	7.64±4.25	1.795	0.057*
MVV (% pred)	48.03±19.2	57.88± 16.3	9.85±3.26	-3.018	0.003*

## Discussion

The present study was undertaken to assess the different psychological aspects and sleep quality of tuberculosis patients and their family caregivers. Sleep disruption may involve a variety of phenomenological entities ranging from sleep disturbances to disrupted or delayed sleep. Various diseases and increased all-cause mortality tend to arise from longer-term sleep deprivation. Many studies have reported that the majority of TB patients suffer from poor sleep quality. The reasons associated with sleep deprivation in such patients are multifactorial, including respiratory symptoms, obstructive sleep apnea, psychiatric disorders, and medication-related insomnia. However, in family caregivers, the factors are more commonly the shift work, emotional stress, disrupted sleep, and stigma to catch the infection themselves that lead to restricted sleep duration and sleep fragmentation [1, 14, 15]. Chronic deprivation of sleep rather than acute lack of sleep may lead to depression that is potentially attributable to the neurochemical changes in the brain. Depression, on the other hand, can lead to deprivation of sleep that may manifest as a symptom of a mood disorder [16]. Short sleep duration has been shown to be increasing in prevalence worldwide with a concurrent increase in depressive symptoms, mainly among the population with chronic diseases [17]. Epidemiological reporting of sleep disturbance in TB patients is limited and in our study, the cause of poor sleep quality among the TB patients is not entirely clear. However, it can be indirectly implied that depression and poor quality of sleep are closely linked, and appear to have a bidirectional relationship where sleep deprivation exaggerates depression and vice versa [18].

Damage to bronchi results from extensive fibrosis or endobronchial stricture as tuberculous sequelae cause airflow obstruction [19, 20]. TB patients account for a significant portion of those with chronic airflow obstruction, and hence in order to evaluate lung function and related clinical issues in patients who suffer from TB, we have compared the various lung function parameters of TB patients with that of their non-TB family contacts. In our study, a decrease in mean FVC and FEV1 among TB patients was evident when compared to their family contacts. Similar studies conducted in Dar es Salaam and elsewhere shows a reduction in FVC, in FEF, PEFR, MVV functions in TB patients when compared to their non-TB family contacts. These studies also concluded that TB patients with a longer duration of TB have a very highly significant reduction in their FVC [21, 22]. However, the FEV1/FVC ratio did not show any significant difference among TB patients when compared with their family contacts. FEVI/FVC ratio is a more sensitive indicator of airway obstruction than FVC or FEV1 alone [23].

Limitations

This cross-sectional study was undertaken to examine the quality of sleep and pulmonary functions through spirometry in TB patients and their non-TB family caregivers at designated treatment sites and hence could not determine the predictors of poor quality of sleep. Although the sample sizes were moderate, they were adequate for comparisons within the site. Clinical samples were selected to represent the patient population of the well-functioning TB control programs at the study site, but they were not intended to represent a profile of TB for the entire country.

## Conclusions

The present study shows that TB patients have a higher prevalence of poor quality of sleep and impaired pulmonary function than their family caregivers. From the overarching results of the study, it can be safely said that there is a possibly well-knit association between tuberculosis and sleep deprivation. This complex association, when unperceived, could result in poorer prognoses of TB cases. There is a consensus in primary care practice that to reduce the associated psychological distress among TB patients and family caregivers, efforts to prevent and manage co-morbidities at the earliest are most needed. In this regard, our study findings have important implications for primary care physicians to carry out a psychiatric evaluation of the TB patients at least once for better treatment outcomes.
